# Enhanced erosion resistance and radiation stability of WC–WO_3_ nanocomposite films under 100 keV Kr^+^ ion irradiation

**DOI:** 10.3762/bjnano.17.65

**Published:** 2026-07-22

**Authors:** Shristi Bist, Parswajit Kalita, Ratnesh K Pandey, Sejal Shah, Richa Krishna, Umapathy G. R., Sunil Ojha, Devesh K Avasthi

**Affiliations:** 1 Department of Physics, School of Engineering, UPES, Dehradun 248007, Uttarakhand, India; 2 ITER India, Institute for Plasma Research, Bhat, Gandhinagar 382428, India; 3 Homi Bhabha National Institute (HBNI), Anushaktinagar, Mumbai 400094, India; 4 Amity Institute of Nanotechnology, Amity University, Noida 201301, India; 5 Inter University Accelerator Centre, New Delhi 110067, India; 6 Centre for Interdisciplinary Research and Innovation, UPES, Dehradun 248007, Uttarakhand, India

**Keywords:** low energy ions, plasma-facing materials, sputtering erosion, WC–WO_3_ nanocomposites

## Abstract

Plasma-facing components (PFCs) in fusion reactors are exposed to extreme radiation and thermal environments, leading to material degradation processes such as sputtering erosion and amorphization. This study investigates and compares the erosion behaviour of tungsten (W) in potential ceramic-based PFC materials, specifically tungsten carbide (WC) thin films and their nanocomposite counterpart, WC–WO_3_, under 100 keV Kr^+^ ion irradiation. The films were irradiated at fluences of 1 × 10^16^, 3 × 10^16^, and 1 × 10^17^ ions/cm^2^. Rutherford backscattering spectrometry was employed to evaluate W sputtering yields and compositional stability. The WC–WO_3_ nanocomposite exhibited significantly reduced W erosion compared to pure WC films, attributed to the higher W binding energy and the existence of heterointerfaces within the nanocomposite matrix. SRIM simulations corroborated the experimental sputtering trends. Furthermore, preferential sputtering of lower-*Z* elements (C and O) was observed in both material systems. Glancing incidence X-ray diffraction analysis revealed post-irradiation grain growth in WC–WO_3_ films, while scanning electron microscopy results show morphological changes, indicating dynamic microstructural evolution rather than amorphization under irradiation. These results highlight the potential of WC–WO_3_ nanocomposites as erosion-resistant and structurally resilient candidates for future plasma-facing applications in fusion reactors.

## Introduction

Tungsten carbide (WC) and tungsten trioxide (WO_3_) are two hard and high-temperature-stable compounds that have individually demonstrated exceptional properties, including high melting points, mechanical strength, and chemical stability [1–5]. WC is a refractory ceramic compound known for its exceptional hardness, high melting point, and chemical inertness [6]. As a ceramic, WC exhibits strong covalent and ionic bonding, contributing to its mechanical strength and thermal stability under extreme conditions. Its unique combination of hardness and resistance to wear, corrosion, and radiation makes WC a critical material for advanced structural and protective coating applications, including cutting tools, wear-resistant coatings, and plasma-facing components in fusion reactors [7–9]. It is being investigated for application as a material for plasma-facing walls [10–11] in fusion reactors due to its extraordinary properties including its high thermal conductivity and mechanical strength [7,12]. The ceramic nature of WC contributes to its stability under such conditions, and recent studies have confirmed its resilience to ion irradiation, reinforcing its potential as a radiation-resistant material in nuclear environments [13]. WO_3_, in contrast, has other advantages, including its capability to act as a radiation absorber and its chemical inertness [14–16]. However, hardness and toughness of the conventionally made coarse-grained cemented carbides are mutual restraints, significantly restricting its uses and applications [17–18]. Additionally, there are other challenges of these materials in large-scale manufacturing, commercialization, and industrial applications [17–20].

Composites have drawn attention owing to their improved mechanical properties, enhanced activities and chemical stability [21–24]. In the recent past, many studies have been carried out which establish synthesis–structure–property relationships of polymer matrix composites in various areas of applications like aircrafts, satellites, automobiles, and sporting goods [25–28]. The potential of utilizing composite materials for efficient shielding/protection and enhanced radiation tolerance has also been explored lately [29–43]. Plasma-facing structural materials are subjected to (micro)structural damage and erosion due to the impact of energetic particles (e.g., hydrogen, helium, neutrons) [44–46]. In earlier decades of fusion research, graphite was explored as a potential plasma-facing component (PFC) material, but it produced mediocre plasma performance with large centre radiation losses leading to several limitations on plasma operations [47–49]. Researchers then started exploring tungsten as a promising PFC material due to its exceptional qualities such as high melting point, high thermal conductivity, and high sputtering threshold energy [50–51]. However, its stability under high-temperature conditions was a matter of serious concern because of its high ductile-to-brittle transition temperature (DBTT) [52]. A study by Baker et al. threw light at the challenges faced in case of using tungsten as a PFC material [53–54]. It has a tendency to form He bubbles and get fragmented; this contaminates the plasma, compromising the purity and efficiency of the fusion plasma. Erosion of the material is one of the major concerns in selection of a PFC material along with the radiation damage studies for nuclear reactor applications. Sputtering causes degradation of the PFC material, and the sputtered atoms contaminate the plasma of tokamaks, which is undesirable. The erosion (sputtering) of films is known to be dependent on several factors such as ion energy [55], composition of material and its properties [56–57], film thickness [58], and substrate [55]. Given its importance, understanding of radiation tolerance has been a topic of long-standing research [59–67]. However, the studies performed till date have almost unilaterally focussed on the radiation-induced changes in single-component materials. A recent study by Ohtaki et al. reported the superior radiation resistance of a composite compared to a single-component material [33]. The enhanced radiation resistance of the composite was attributed to the presence of heterointerfaces (in the composite) that act as better sinks for the irradiation induced defects as compared to grain boundaries in the single-component material [33,68]. Some recent studies of ion induced effects in composite materials are tabulated in Table 1.

**Table 1:** Ion irradiation studies of composite materials.

No.	Title	Sample	Incident ion	Observations

1.	Improved high temperature radiation damage tolerance in a three-phase ceramic with heterointerfaces [33]	Ceramic composite system containing equal portions of Al_2_O_3_, YSZ and MgAl_2_O_4_	4 MeV Si^2+^ at 650 °C	The concentration of dislocation loops in a radiation damage-prone phase (Al_2_O_3_) is significantly reduced when Al_2_O_3_ is in a composite system as opposed to a single-phase system.
2.	Influence of Ni^13+^ ions irradiation on the microstructure, mechanical and tribological properties of Mo-S-Ti composite films [34]	Mo-S-Ti composite films	2 MeV Ni^13+^	The Ni^13+^ irradiation has less effect on the Mo-S-Ti composite films surface, but caused an observable damage to the inner of the thin film, and the severely damaged area due to irradiation is mainly in the range of 1.13–1.88 μm. The hardness and elastic recovery ratio of the film after irradiation were enhanced up to 12.74 GPa and 65%, respectively.
3.	Effect of medium energy He^+^, Ne^+^ and Ar^+^ ion irradiation on the Hf-In-C thin film composites [35]	Hf-In-C thin film composites	100 keV He^+^, 100 keV Ne^+^, 200 keV Ar^+^	Hf-In-C thin films exhibit high radiation resistivity to He^+^ ions. There were no significant changes in the structure or surface morphology after irradiation even at highest fluence of 10^17^ ions/cm^2^. However, for irradiation with Ne^+^ and Ar^+^ ions, severe damage in Hf-In-C films was observed. The most significant change was a considerable decrease in the content of In and C building elements.
4.	Mo-S-Pb-Ti amorphous films: Achieving exceptional irradiation tolerance and enhanced wear resistance [36]	Mo-S-Pb-Ti composite films	2 MeV Au^2+^	The prepared composite film successfully realizes a combination of anti-irradiation and wear resistance of MoS_2_-based composite films. Mo-S-Pb-Ti composite films exhibit a distinctly different radiation response compared to MoS_2_ films under ion irradiation. Upon irradiation, a nano-multilayer structure was spontaneously formed in irradiated regions, which demonstrates extremely high radiation resistance.
5.	Hydrogen isotope retention in plasma-facing materials: review of recent experimental results [37]	W and carbon fibre composites (CFCs)	200 eV D^+^	CFCs retain the highest levels of hydrogen and erode quickly. Retention in W depends strongly on the material structure. The total D^+^ retention in single- and polycrystalline W increases with the exposure temperature.
6.	Comparison of D retention for advanced plasma facing materials by D ion implantation [38]	W-Ta, W-Mo and K-doped W	1–3 keV D_2_^+^	No large D retention enhancement was found. Particularly, the formation of large voids was refrained and was the major D trapping sites for K-doped W.
7.	A candidate fusion engineering material, WC-FeCr [39]	WC-FeCr composites	6 keV He^+^ at RT and 500 °C	The FeCr phase grew a coarse array of bubbles, which underwent preferential growth at 500 °C. The WC phase formed a much finer array of uniformly sized bubbles that were stable at both irradiation temperatures. Spherical-cap bubbles were observed at WC/M_6_C and WC/Cr-C interfaces, which were much larger than on WC/FeCr interfaces.
8.	Copper matrix composites as heat sink materials for water-cooled divertor target [40]	CuCrZr, W-Cu, SiC-Cu nanocomposites	Neutron irradiation (1–10 displacements per atom)	The three composites exhibited far superior ultimate tensile strength at elevated temperatures. The benefits of Cu matrix composites for the heat sink application are enhancement of higher-temperature strength and reduction of thermal stress by mitigating the thermal strain mismatch.
9.	Carbon fiber composites application in ITER plasma facing components [41]	CFCs	Neutron irradiation (≤1 displacements per atom)	Demonstration of excellent thermomechanical performance of CFC-armoured mock-ups and the absence of melting giving higher erosion lifetime.
10.	Radiation effects in tungsten and tungsten-copper alloys treated with compression plasma flows and irradiated with He ions [42]	W-Cu composites	40 keV He^2+^	Increased radiation resistance of samples pre treated with CPF in the melting and mixing mode of the near surface region. The increased number of intergranular boundaries mitigates the effect of surface destruction due to the migration of radiation defects to the boundaries.
11.	Neutron irradiation induced elemental activation and its effect on phase stability of Al_2_O_3_-YSZ composite [43]	Al_2_O_3_ YSZ composites	14 MeV neutrons	Neutron exposure resulted in formation of ^24^Na which is responsible for the longer activity of Al_2_O_3_, whereas formation of ^88^Y, ^90^Y, and ^89^Zr, which are slowly decaying nuclides, contribute to the prolonged activity of YSZ in composites. Doping YSZ in alumina improves structural stability of the composite.

From the extensive literature review discussed in Table 1, it can be seen that none of the studies have explored ion-induced erosion in the composite thin films. Thus, the present work aims to investigate the erosion behaviour of WC–WO_3_ nanocomposite films and its comparison with WC films under “low-energy” ion irradiation, i.e., in the nuclear energy loss regime. It is important to note that the nuclear energy loss (*S*_n_) resulting from ion irradiation in this study is significantly greater than the anticipated energy loss from lighter energetic particles typically expected in a fusion reactor. Here, 100 keV Kr^+^ ions (nuclear energy loss, *S*_n_ = 303.2 eV/Å; electronic energy loss, *S*_e_ = 60.59 eV/Å) depositing a higher energy density to the material are being considered to test the behaviour of synthesized nanocomposite films under far more extreme irradiation conditions. Numerous studies on the effect of Kr^+^ ion irradiation across different materials have demonstrated a wide range of ion-induced modifications in both surface and bulk of the materials, with the nature and extent of these changes strongly dependent on the ion fluence [69–73]. In one such work by Gan et al. [73], the stability of five different phases of depleted uranium alloys under Kr^+^ ion irradiation was studied to investigate the irradiation-induced damage to the microstructure from fission up to 10 dpa (displacements per atom). The novelty of the present study lies in the fact that no other research group has explored the effects of ion irradiation on the WC–WO_3_ composite material to the best of our knowledge. Additionally, no one has studied sputtering due to ion irradiation in case of composite films. The findings of this study will contribute to the existing database of alternative radiation-resistant materials, beyond tungsten, for potential applications in nuclear reactor technologies.

## Materials and Methods

The WC films were synthesized by sputter deposition on Si substrate heated at 600 K. Before the deposition, the substrates were sonicated for 10 min and then cleaned thoroughly using acetone. The sputter deposition chamber had a base vacuum of 4.9 × 10^−6^ mbar before the deposition was obtained using a turbomolecular pump backed by a rotary pump. The deposition was done under 3 × 10^−2^ mbar pressure with a power of 150 W RF for 30 min in the presence of argon gas having flow rate of 30 sccm. The WC–WO_3_ nanocomposite films were synthesized using sputter deposition of WC films at room temperature (RT) and its subsequent annealing at ~700 °C in a tubular furnace in air atmosphere. The oxygen in air facilitates the growth of the carbide and oxide phases simultaneously at high temperature. Otherwise, the as-deposited films were amorphous as shown in Figure 2a. A description of the synthesized samples of typical sizes of 1 cm × 1 cm, namely S1 (WC–WO_3_ nanocomposite films) and S2 (WC films) is given in Table 2.

**Table 2:** Sample details.

Sample name	Description

S1	WC–WO_3_ nanocomposite films (700 °C annealed RT deposited WC films)
S2	WC films (deposited at 600 K substrate temperature)

**Figure 1:**
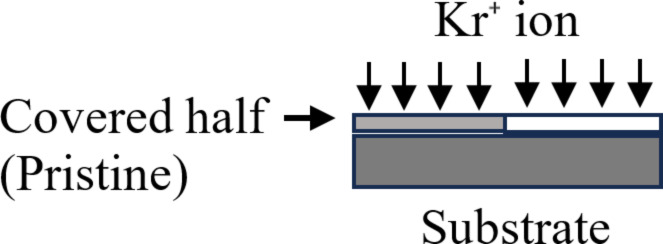
Schematic of the cross-sectional view of the half-irradiated films.

Later, half part (0.5 cm × 1 cm) of both the samples were irradiated with 100 keV Kr^+^ ions, at normal incidence, from ECR ion source based low energy ion beam facility (LEIBF) at Inter University Accelerator Centre (IUAC), New Delhi. In case of S1, the irradiations were performed at three fluences viz. 1 × 10^16^, 3 × 10^16^, and 1 × 10^17^ ions/cm^2^, while for S2, the irradiation was performed at the highest fluence of 1 × 10^17^ ions/cm^2^. During the irradiation one half of the films was covered with aluminium foil, such that only the other half of the film is exposed to ion irradiation keeping the covered half pristine, as shown schematically in Figure 1, to determine the sputter yield using Rutherford backscattering spectrometry (RBS) measurements post-irradiation. S1 samples were also irradiated by scanning the complete area (i.e., 1 cm × 1 cm) to study the structural modifications due to ion irradiation.

The pristine and irradiated halves were analysed using RBS with a 2 MeV He-ion beam from the 1.7 MV Pelletron accelerator at IUAC to measure the sputtering/erosion rate. The RBS measurements were taken at a scattering angle of 165°, with a beam spot size of around 2 mm in diameter. The RBS spectra were analysed using SIMNRA 6.06 software [74].

Further, to find the crystalline state of the nanocomposite films (S1) both before and after irradiation, glancing incidence X-ray diffraction (GIXRD) measurements were performed. The measurements were carried out in a D-8 X-ray diffractometer (procured from Bruker AXS Germany) in the 2θ range from 10° to 60°. GIXRD measurements of the pristine S2 films were also performed; these measurements were carried out in an Empyrean X-ray diffractometer (procured from Panalytical, Netherlands) in the 2θ range from 20° to 80°. Scanning electron microscopy measurements of pristine and irradiated S1 films were performed using a MIRA Tescan II SEM at a high voltage of 25 kV using detector working in secondary-emission mode.

## Results and Discussion

### Phase and compositional studies of the pristine WC–WO_3_ nanocomposite and WC films

**Figure 2:**
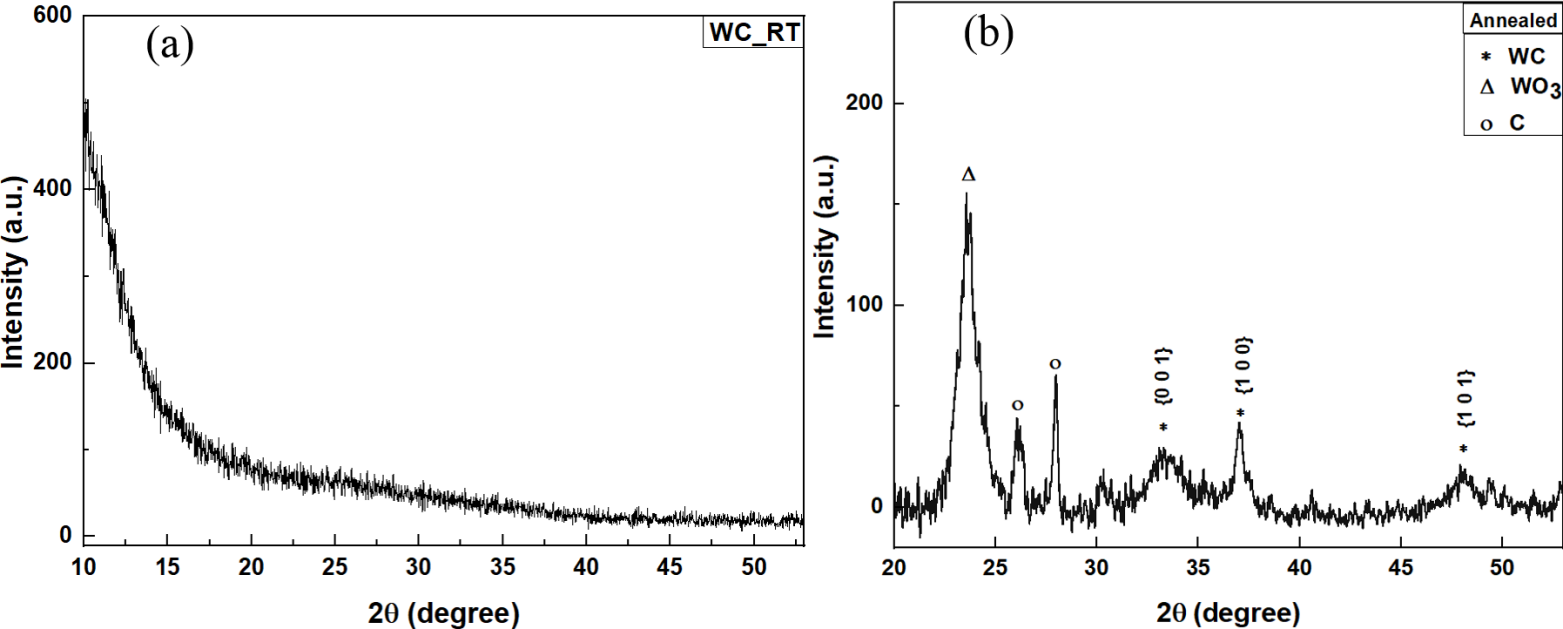
GIXRD pattern of (a) as-deposited (WC_RT) and (b) 700 °C annealed (S1) films in air ambient.

The GIXRD pattern of the as-deposited WC films, shown in Figure 2a, reveals that the room-temperature-deposited films were amorphous as no reflection peak was observed in the pattern. However, after thermal annealing of these films at ~700 °C, grain growth took place and a carbide–oxide nanocomposite of tungsten was formed, which is evident in Figure 2b. The polycrystalline phases of the annealed films were observed showing reflections at 2θ values of 33.49°, 36.95°, and 48.16° corresponding to (001), (100), and (101) planes of WC, respectively (JCPDS-96-150-1517). Further, an intense peak of tungsten oxide was observed at 23.50° corresponding to the (001) plane of WO_3_ (JCPDS-96-210-1051).

**Figure 3:**
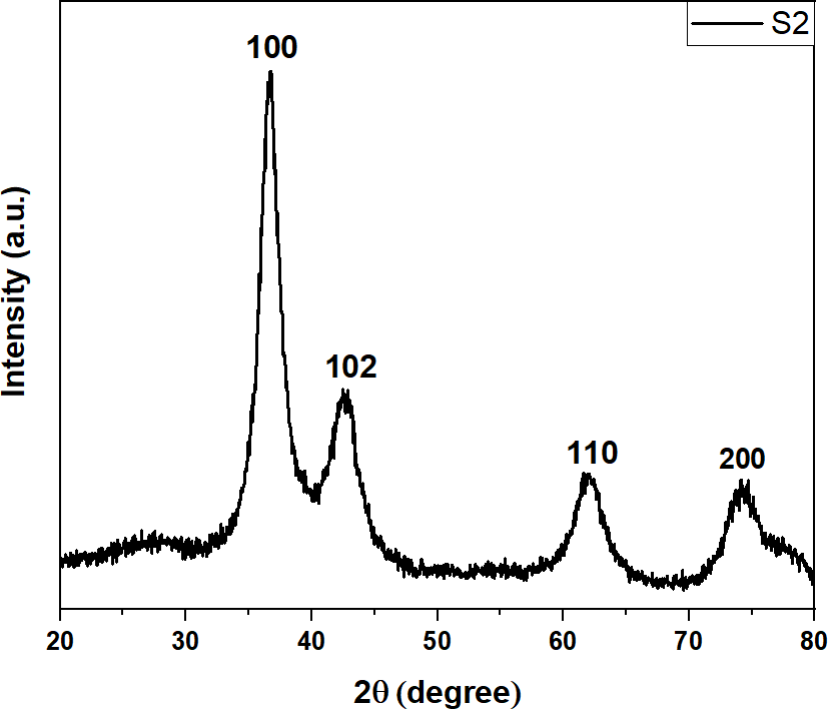
GIXRD patterns of WC films (S2) deposited at 600 K substrate temperatures.

The GIXRD patterns of the S2 films are displayed in Figure 3. These measurements indicate the polycrystalline nature of the films as the reflections observed at 2θ values of 36.81°, 62.05°, and 74.4°, which correspond to the (100), (110), and (200) planes of WC, respectively (JCPDS-96-150-1543). Additionally, a reflection at 42.54° corresponds to the (102) plane of W_2_C (JCPDS-96-153-9793).

The grain size (*D*) of the samples was determined using the Scherrer formula, 
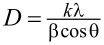
, with *k* being a constant (shape factor; *k* = 0.9), λ the wavelength of the X-rays, β the full width at half maximum (FWHM) of the peak (in radians), and θ the Bragg angle (in degrees). The calculations showed that the S1 film had a grain size of approximately 17.5 nm, while the S2 film had a grain size of around 6 nm.

**Figure 4:**
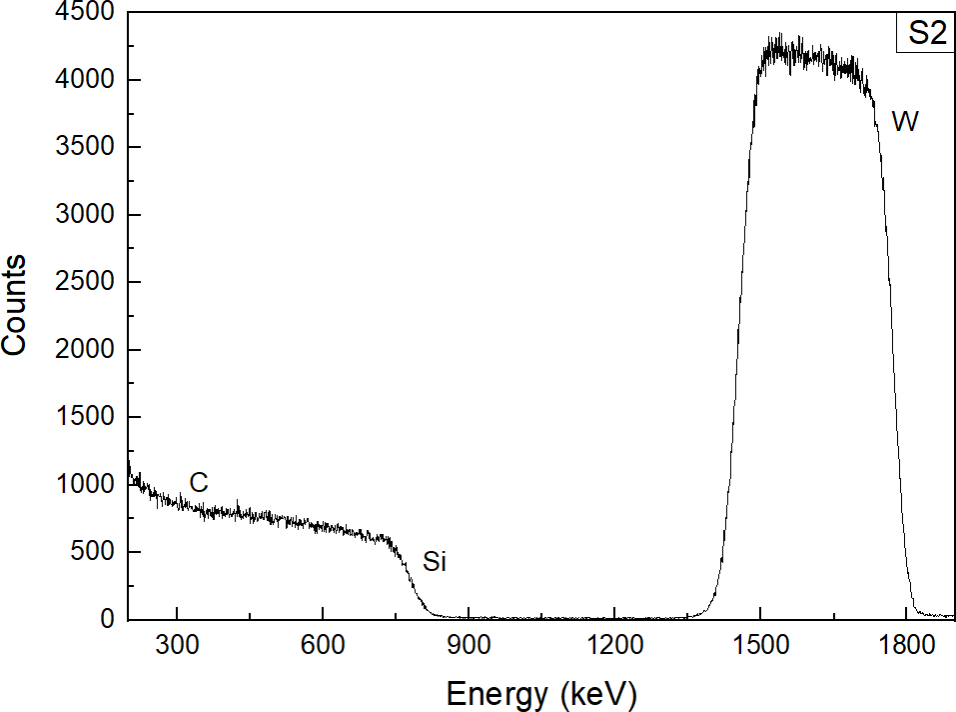
Typical RBS spectra of S2 films.

Figure 4 displays a typical RBS spectrum of the WC films (S2). The peak at higher energy values represents tungsten (W). The carbon (C) peak was not clearly distinguishable due to carbon’s small atomic number and low Rutherford scattering cross section. The continuous broad region at lower-energy values corresponds to silicon (Si). Using SIMNRA 6.06 software after fitting the experimental data, the stoichiometry of W and C in the sample was determined to be roughly 1:1, while the thickness of the film was found to be ~400 nm.

### Determination of sputtering in the films

**Figure 5:**
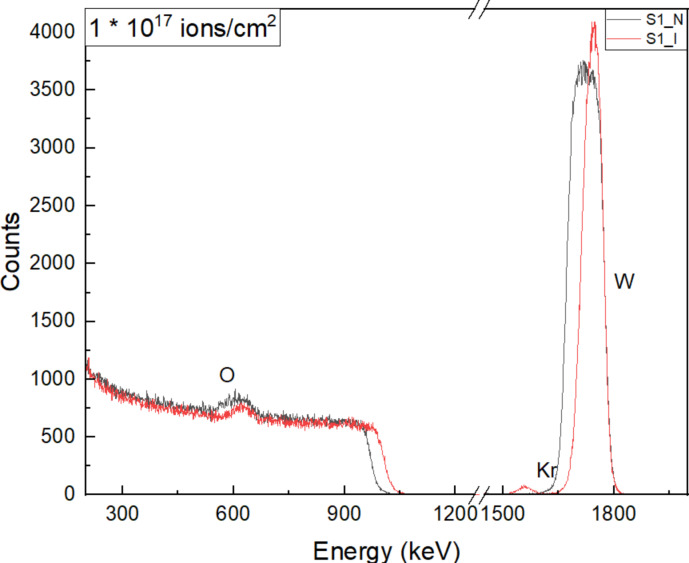
RBS spectra of the S1 films – pristine (black; S1_N) and irradiated (red; S1_I) at fluence 1 × 10^17^ ions/cm^2^.

**Figure 6:**
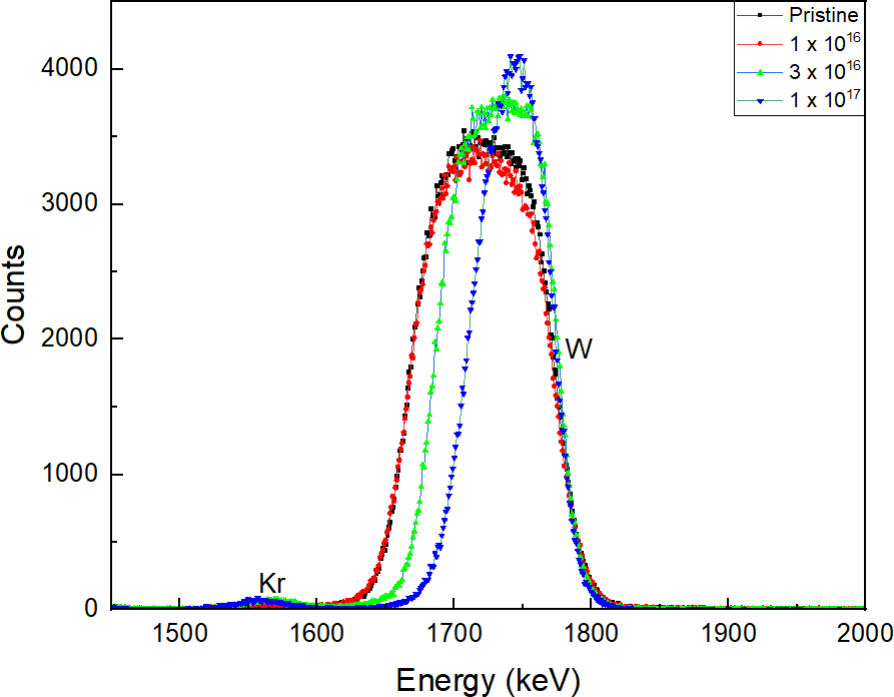
RBS spectra of the W peak of the S1 films – pristine (black) and irradiated with 100 keV Kr^+^ ions at fluence 1 × 10^16^ ions/cm^2^ (red), 3 × 10^16^ ions/cm^2^ (blue) and 1 × 10^17^ ions/cm^2^ (green).

The RBS spectra of pristine and irradiated (at fluence at 1 × 10^17^ ions/cm^2^) regions of the S1 film are shown in Figure 5. The RBS data acquired from the covered half during the irradiation was considered pristine. The backscattered spectra of pristine and irradiated regions of the S1 films at different fluences obtained for the W peak of the RBS spectra are shown in Figure 6. It is clear from the spectra that the increase in ion fluence caused a reduction in the width of the W peak and reduction in the area of the W peak, indicating sputtering. At a fluence of 1 × 10^16^ ions/cm^2^, both width and intensity of W peak were slightly less than those of the pristine sample, while for higher fluences the width decreased but the height of the peak increased.

**Figure 7:**
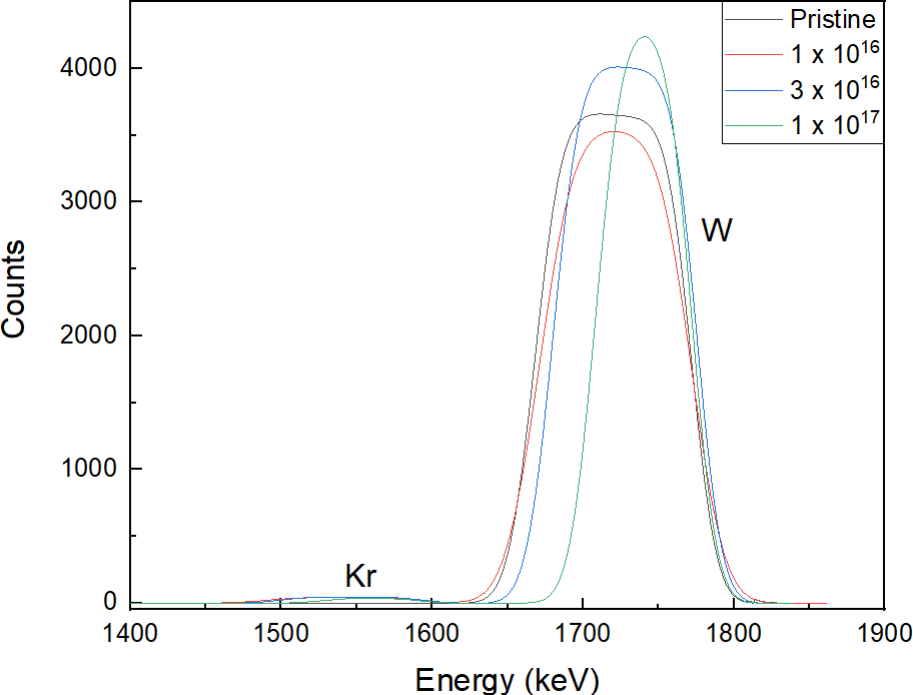
Simulated spectra of W peak (S1 films) using SIMNRA of Pristine (black) and 100 keV Kr^+^ ion irradiation at fluences (ions/cm^2^): 1 × 10^16^ (red), 3 × 10^16^ (blue), and 1 × 10^17^ (green).

We performed SIMNRA simulations by fitting the experimentally obtained data for a quantitative analysis of these observations. The W peaks of the resulting simulated spectra for pristine and irradiated films are shown in Figure 7, and the areal concentrations of the elements are given in Table 3.

**Table 3:** Areal concentrations obtained from SIMNRA fitting of the pristine and irradiated WC–WO_3_ nanocomposite (S1) films.

	Areal concentration (× 10^15^ atoms/ cm^2^) of S1 samples			
	W	C	O	Total

pristine	220.5	418.0	451.0	1089.5
1 × 10^16^ ions/cm^2^	209.0	407.0	440.0	1056.0
3 × 10^16^ ions/cm^2^	191.1	392.0	377.0	960.1
1 × 10^17^ ions/cm^2^	156.0	246.0	180.0	582.0

It is apparent from Table 3 that there is initially (at 1 × 10^16^ ions/cm^2^) a slight reduction in the W, C, and O content, indicating the sputtering of the atoms from the films. The areal concentration decreased significantly in the case of irradiation at the higher fluences (i.e., at 3 × 10^16^ ions/cm^2^ and 1 × 10^17^ ions/cm^2^). This indicates stronger sputtering of the atoms from the films upon irradiation, causing a reduction in the width of the W peak (Figure 6). However, interestingly, the relative decrease in W concentration is less than that of C and O, indicating a preferential (i.e., intensified) sputtering of the low-*Z* elements C and O, which is manifested by increased intensity of W peak at higher fluences (Figure 6). Similar accounts of preferential sputtering of lighter atoms due to energetic ion irradiation has been observed by other researchers as well [75–78].

**Figure 8:**
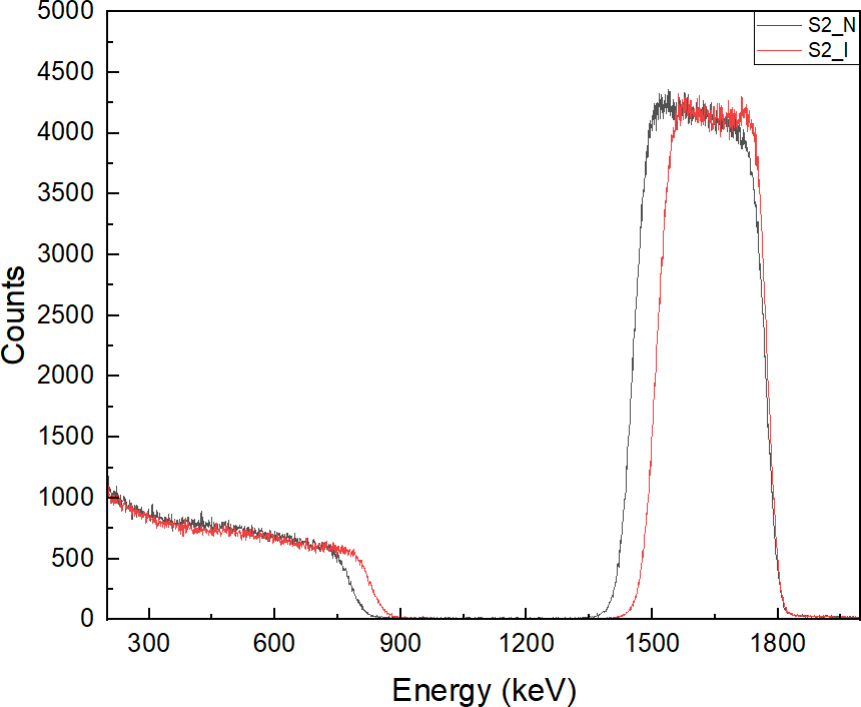
RBS spectra of WC films (S2). The black curve is for the pristine (S2_N) and the red curve is for the irradiated (1 × 10^17^ ions/cm^2^; S2_I) half of the sample.

The experimental RBS spectra for the pristine and irradiated, S2 film is shown in Figure 8. A closer look of the spectra yields the following observations similar to the previous case: (i) The reduction in the width of the W peak post-irradiation signifies the sputtering of tungsten due to ion irradiation. (ii) A distinctive rise in the W peak height at energy values around 1800 keV post-irradiation indicates a preferential sputtering of carbon.

**Figure 9:**
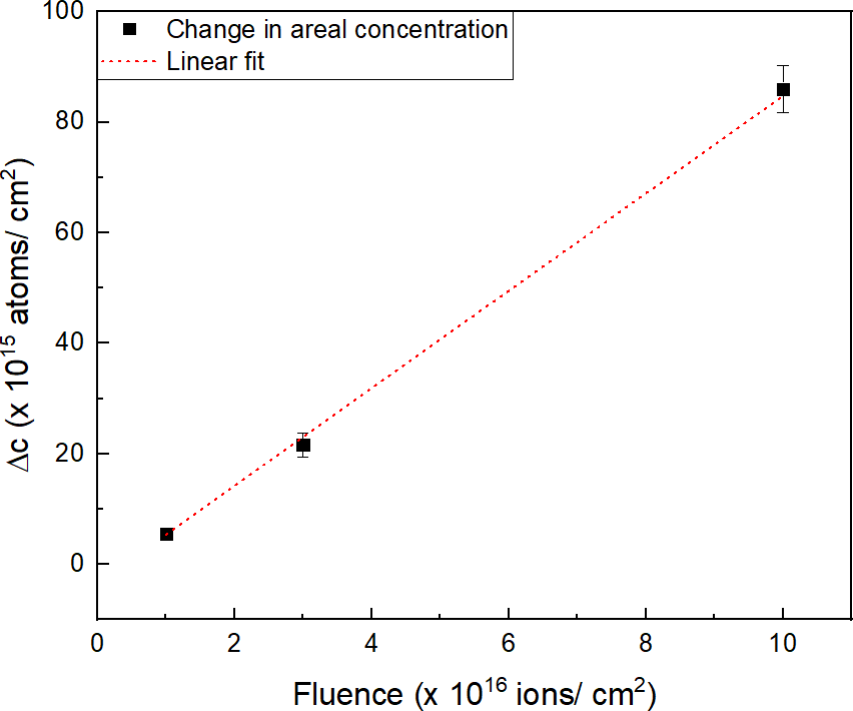
Variation in differential areal concentration with ion fluence for S1. The sputter rate is obtained from the slope of this curve.

For a quantitative estimation of the irradiation induced sputtering and/or erosion in the S1 and S2 films, the sputter rate was obtained using the following relation [55,79]:


[1] $$\text{sputter rate}=\frac{\text{change in areal concentration}}{\text{change in fluence}}=\frac{\Delta c}{\Delta \phi },$$

where *∆c* is the difference in the areal concentration of W in the pristine and irradiated halves of the film (considering the W peak as shown in Figure 6 and Figure 8). For the S1 film, the differential areal concentration has been plotted as a function of the fluence (Figure 9). The slope of this plot thus gives the sputter rate of W, which in this case is ~0.9 atom/ion. In contrast, the sputtering rate of W in WC films irradiated with 100 keV Kr^+^ ions, as determined using Equation 1, was found to be approximately 3.2 atoms/ion. This indicates higher sputtering of W in WC films compared to WC–WO_3_ nanocomposite films. Table 4 shows the comparision of sputter rate in the samples S1 and S2 estimated from SIMNRA simulations.

**Table 4:** Comparison of sputter rate in samples S1 and S2 as estimated from SIMNRA simulations.

No.	Sample	Experimental sputter rate of W (atoms/ion)

1	S1	0.88 ± 0.03
2	S2	3.20 ± 0.01

Theoretical calculations using TRIM [80] simulations yielded sputtering rates of ~4.5 atoms/ion for WC and ~1.9 atoms/ion for WC–WO_3_, consistent with the experimental trend of higher sputtering in WC films. However, the theoretical values are slightly higher than those derived experimentally using RBS data fitted via SIMNRA. Such deviations of experimental and theoretical values have been reported earlier also [81–82]. The difference between the experimentally determined values of sputtering and theoretical predicted values can be explained as follows: The sputtering process induced by ion–solid interactions can be theoretically be understood through Sigmund’s theory of sputtering [83–84] within the framework of the “linear cascade” regime. Sigmund’s formula [83] is given as:


[2] $$Y=\frac{0.042\alpha S_{\text{n}}\left(E_{0}\right)}{U},$$

where *Y* is the sputtering yield, *U* is the surface binding energy, α is a function of mass ratio of target and ions, *S*_n_(*E*) is the stopping power of ions in the target at energy *E*, and *E*_0_ is the initial energy of the ions. The deviation between experimental and TRIM results for sputtering could be a result of non-linear effects. TRIM assumes a simplified energy transfer model, ignoring oscillatory variations in stopping power that arise due to the specific interaction between the ions (*Z*_1_) and the target atoms (*Z*_2_). The energy loss may be lower than the predicted TRIM values because energy transfer efficiency changes for certain *Z*_1_ and *Z*_2_ combinations where resonance-like effects are absent. This results in TRIM overestimating/understimating sputtering yields compared to experimental data, and deviations upto 30% have been reported previously [81–82].

The lower erosion (i.e., greater stability) of WC–WO_3_ nanocomposite films relative to the pure WC films is attributed to the binding energy values of individual components. Binding energy values in the W 4f region for WC, and WO_3_ are 31.3–31.8 eV (7/2), 33.9 eV (5/2) for WC and 35.5 eV (7/2), 37.7 eV (5/2) for WO_3_ [85–86]. The higher binding energy of W in WO_3_ as compared to WC suggests that the formation of the WC–WO_3_ nanocomposite results in increased overall binding energy, enhancing stability. The relatively low binding energy of WC as compared to that of WO_3_ facilitates the easier displacement of W atoms under ion bombardment, explaining the higher sputtering rate in WC films than that in nanocomposite films.

Another explanation for the lower erosion of the nanocomposite films could be potentially attributed to the presence of heterointerfaces, which are better sinks for radiation-induced interstitials/vacancies than grain boundaries [33]. The transfer of energy from the incident ion(s) results in the displacement of the target atoms from their lattice sites in collison cascades, and may even ultimately lead to their ejection from the material itself (i.e., sputtering). Given the ability of heterointerfaces (in composites) to accommodate a higher concentration of interstitials/vacancies than grain boundaries in single-component materials [33], there is a possibility that a higher number of displaced atoms are accomodated in the composite by the heterointerfaces, resulting in a smaller number of displaced atoms being ejected from the surface. Note that differences in chemical potential in composites can further influence the migration of interstitals/vacancies to heterointerfaces [33,87]. However, studies on this hypothesis are very limited, and further research is needed to draw a definite conclusion on the role of heterointerfaces in lowering the erosion of the nanocomposite films by taking different nanocomposites.

From the above discussion it is clear that erosion in nanocomposite samples is less than in pure WC samples. Therefore, it is of interest to see the structural and morphological changes in the nanocomposite samples under low-energy ion irradiation; this is discussed in the following section.

### Structural and surface modifications in nanocomposite films upon Kr^+^ ion irradiation

**Figure 10:**
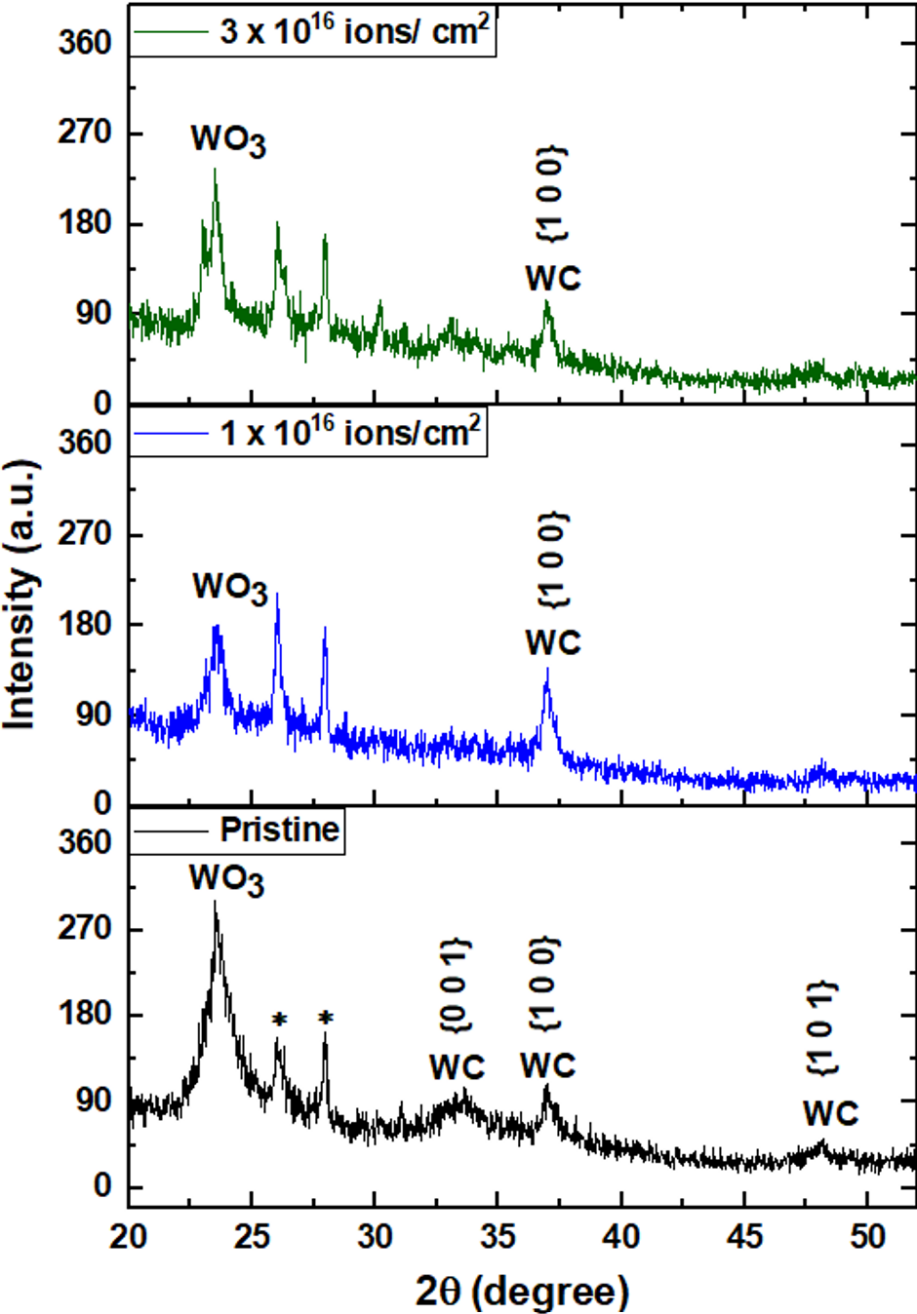
GIXRD patterns of pristine and irradiated WC–WO_3_ nanocomposite films (S1) at different fluences. The asterisks represent carbon peaks.

The GIXRD patterns of the S1 films irradiated with 100 keV Kr^+^ ions at different ion fluences are shown in Figure 10. It is apparent that there are no major structural modifications upon irradiation. A closer analysis however indicates irradiation-induced grain growth in the films. Similar post-irradiation grain growth has been reported earlier by other researchers for different materials [88]. Considering the (100) peak of WC, the grain sizes are found to be 17.5, 18.2, and 19.9 nm, from the Scherrer equation, for the pristine film and films irradiated with ion fluences 1 × 10^16^ and 3 × 10^16^ ions/cm^2^, respectively. Also, considering the (001) peak of WO_3_, the grain sizes are calculated to be 7.9, 11.3, and 10.5 nm for the pristine film and the films irradiated at 1 × 10^16^ and 3 × 10^16^ ions/cm^2^, respectively. These results suggest that irradiation induces grain growth. The grain growth in both the carbide and oxide phases, as determined from the GIXRD patterns, is summarized in Table 5.

**Table 5:** Variation of grain size with fluence for the nanocomposite (S1) films.

Ion fluence (ions/cm^2^)	Grain size (nm)	

	WC	WO_3_

pristine	17.5 ± 1.8	7.9 ± 0.8
1 × 10^16^	18.2 ± 1.9	11.3 ± 1.1
3 × 10^16^	19.9 ± 2.9	10.5 ± 1.1

Low-energy ion irradiation-induced grain growth has been observed in other nanocrystalline systems [61,68], and a defect-driven mechanism typically explains it. This mechanism posits that the accumulation of irradiation-induced point defects, created through collision cascades, leads to mechanical instability in the crystallites. In nanocrystalline materials, collision cascades and subsequent defect formation occur near the grain boundaries. Since grain boundaries act as defect sinks, these defects tend to migrate towards them. In such a setting, the cooperative movement of defects towards the grain boundaries stimulates grain boundary migration. This migration results in overall grain growth through atomic jumps of the defects across the grain boundaries [61,68]. The defect density can be estimated via SRIM/TRIM simulations [80] in terms of displacements per atom (dpa). The formula for damage in units of dpa is [13]:


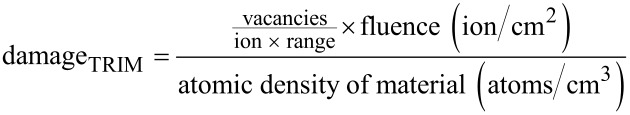


From TRIM simulations, the vacancies/ion generated by 100 keV Kr^+^ ions are ~1210 and ~1435 for WC–WO_3_ (Range: ~35 nm) and WC (Range: ~20 nm) films, respectively. Therefore, the estimated damage in dpa at the highest fluence of 1 × 10^17^ ions/cm^2^ is ~5.4 × 10^2^ for WC–WO_3_ nanocomposite films and ~7.5 × 10^2^ for WC films. The dpa values for WC–WO_3_ nanocomposites and WC also estimate less damage in case of nanocomposite films as compared to the WC films.

**Figure 11:**
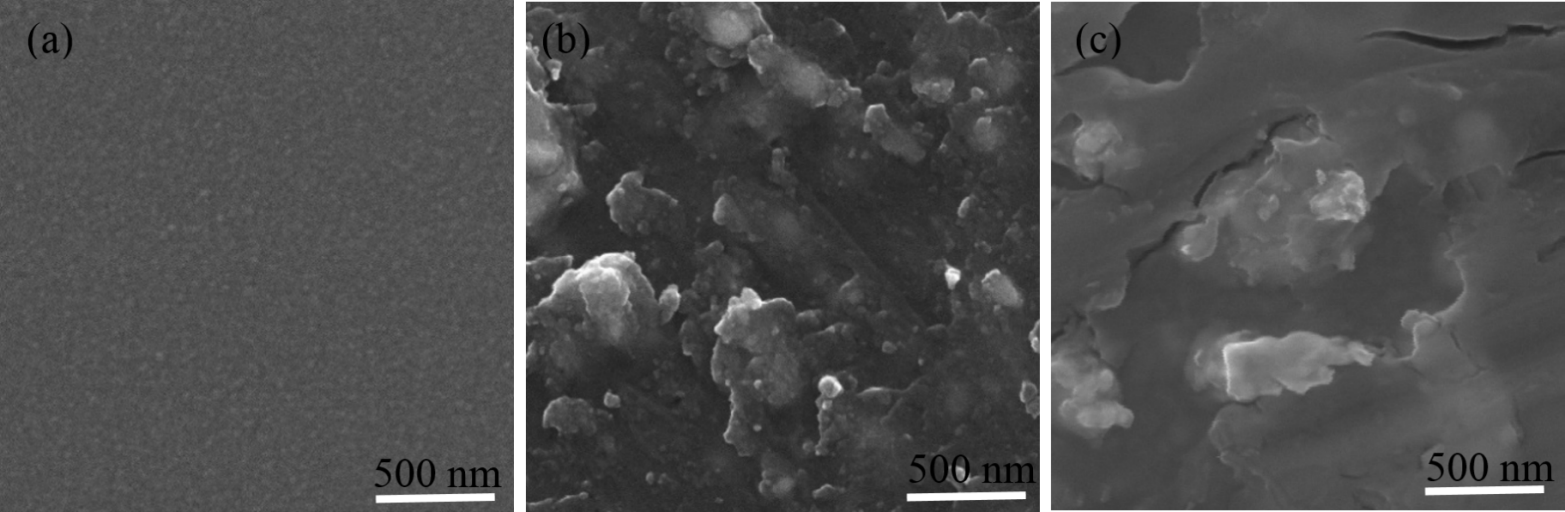
SEM images of (a) pristine and irradiated WC–WO_3_ nanocomposite films (S1) at different fluences: (b) 1 × 10^16^ ions/cm^2^; (c) 3 × 10^16^ ions/cm^2^.

The pristine and irradiated S1 samples underwent SEM measurements to see morphological changes under ion irradiation. The SEM images of the pristine S1 films and films irradiated at ion fluences of 1 × 10^16^ and 3 × 10^16^ ions/cm^2^ are shown in Figure 11.

The SEM images clearly demonstrate significant morphological surface changes in the WC–WO_3_ nanocomposite films induced by ion irradiation. The pristine film exhibits a uniform, densely packed nanograin structure, indicative of a smooth and homogeneous surface. Upon irradiation at a fluence of 1 × 10^16^ ions/cm^2^, the surface becomes increasingly rough, and the formation of distinct nanoclusters is observed. At a higher fluence of 3 × 10^16^ ions/cm^2^, the surface morphology evolves further, showing enhanced clustering and the emergence of surface features such as voids and cracks, suggesting substantial structural modification.

These changes arise from ion–solid interactions, primarily driven by collision cascades initiated by energetic Kr^+^ ions. As ions penetrate the surface, they transfer energy to lattice atoms, causing atomic displacements that lead to the creation of vacancies, interstitials, and localized disorder. At elevated fluences, the cumulative effect of these displacements results in pronounced surface restructuring. Similar phenomena, including ripple formation and surface roughening, have been widely reported and are known to depend on parameters such as ion energy, fluence, and target material properties [89–90].

The SEM and GIXRD results show strong correlation in revealing the microstructural evolution of WC–WO_3_ nanocomposite films under ion irradiation. SEM images indicate progressive surface roughening, nanocluster formation, and morphological degradation with increasing fluence, particularly evident at 3 × 10^16^ ions/cm^2^. The grain coarsening seen in XRD data is consistent with the clustering behaviour observed in SEM micrographs, implying that atomic displacements and defect accumulation during ion bombardment promote local atomic mobility, enabling the reorganization of nanocrystalline domains. Furthermore, the absence of amorphization and the persistence of crystalline features in XRD patterns, even at higher fluences, confirm that the WC–WO_3_ nanocomposite maintains a degree of structural integrity, despite surface modifications. Altogether, these results highlight that while ion irradiation alters surface morphology significantly, the overall crystalline nature of the nanocomposite remains intact, suggesting its suitability for plasma-facing applications where both surface stability and structural robustness are important.

## Conclusion

The present study comprehensively examined the response of nanocrystalline WC and WC–WO_3_ nanocomposite thin films to 100 keV Kr^+^ ion irradiation, with particular emphasis on sputtering behaviour, structural stability, and irradiation-induced microstructural evolution. RBS analysis revealed a significantly lower sputtering yield in case of WC–WO_3_ nanocomposite films (~0.9 atoms/ion) as compared to WC films (~3.2 atoms/ion), demonstrating substantially improved resistance to ion-induced erosion. The reduced sputtering rate in the nanocomposite films is attributed to modification of the effective surface binding energy of W in the composite matrix and the presence of heterointerfaces, highlighting the improved resistance of the WC–WO_3_ system to ion-induced erosion.

GIXRD results confirm that both WC and WO_3_ phases of the nanocomposite film retain their crystalline structure even at higher ion fluences, with no observable amorphization or phase decomposition. Additionally, a moderate increase in crystallite size is observed, suggesting irradiation-induced defect recombination. SEM observations further reveal surface morphological evolution, consistent with irradiation-driven atomic redistribution rather than structural degradation. The correlation between GIXRD and SEM results indicates that ion irradiation promotes localized defect migration and structural rearrangement while preserving overall phase stability.

Overall, the combined findings confirm that WC–WO_3_ nanocomposite films exhibit superior erosion resistance and maintain structural integrity under energetic ion irradiation conditions. As compared to previous studies, this is the first of its kind on a WC–WO_3_ nanocomposite thin film system, and also no previous study has systematically investigated the radiation damage and erosion in nanocomposite films under ion irradiation to the best of our knowledge. The findings of this study highlight the potential of carbide–oxide nanocomposite architectures as a promising candidates for plasma-facing components and other extreme-radiation environments, where resistance to sputtering-induced degradation and microstructural stability are critical.

## Data Availability

Data generated and analyzed during this study is available from the corresponding author upon reasonable request.
